# Supplementary material for: Development of AGT-7: An Innovative 99mTc-Labeled Theranostic Platform for Glioblastoma Imaging and Therapy
Source: Pharmaceuticals (Basel). 2025 Aug 8;18(8):1175. doi: 10.3390/ph18081175 (PMC12388980; doi:10.3390/ph18081175)
Supplement: Supplementary file 1 [file pharmaceuticals-18-01175-s001.zip › pharmaceuticals-3769692-supplementary.pdf]

SUPPLEMENTARY INFORMATION

Development of AGT-7: An Innovative ^{99m}Tc-Labeled Theranostic Platform for Glioblastoma Imaging and Therapy

Stavroula G. Kyrkou [1], Vasileios-Panagiotis Bistas [1], Evangelia-Alexandra Salvanou [2], Timothy Crook[4], Maria Giannakopoulou [3], Vasiliki Zoi [3], Maximos Leonardos [5], Andreas Fotopoulos [3], Chrissa Sioka [3], Ioannis Leonardos[5], George A. Alexiou [3], Penelope Bouziotis [2,*] and Andreas G. Tzakos [1,*]

1 Department of Chemistry, Section of Organic Chemistry and Biochemistry, University of Ioannina, 45110 Ioannina, Greece;
2 Institute of Nuclear & Radiological Sciences & Technology, Energy & Safety, National Center for Scientific Research "Demokritos", 15341 Athens, Greece;
3 Neurosurgical Institute, School of Medicine, University of Ioannina, 45500 Ioannina, Greece;
4 John Fulcher Neuro-Oncology Laboratory, Department of Brain Sciences, Division of Neuroscience, Faculty of Medicine, Imperial College London, London W12 0NN, UK;
5 Laboratory of Zoology, Department of Biological Applications and Technologies, University of Ioannina, 45110, Ioannina, Greece;
* Correspondence:, bouzioti@rrp.demokritos.gr, atzakos@uoi.gr; Tel.: (optional; include country code; if there are multiple corresponding authors, add author initials)

Contents

Purity analysis of compound AGT-7 by analytical HPLC 2
NMR Characterization of tert-butyl (4-(bis(pyridin-2-ylmethyl)amino)butyl)carbamate 3
NMR Characterization of N1,N1-bis(pyridin-2-ylmethyl)butane-1,4-diamine 4
Purification by preparative HPLC of compound AGT-7 5
NMR and MS-Based Characterization of AGT-7 5
References 7

Purity analysis of compound AGT-7 by analytical HPLC

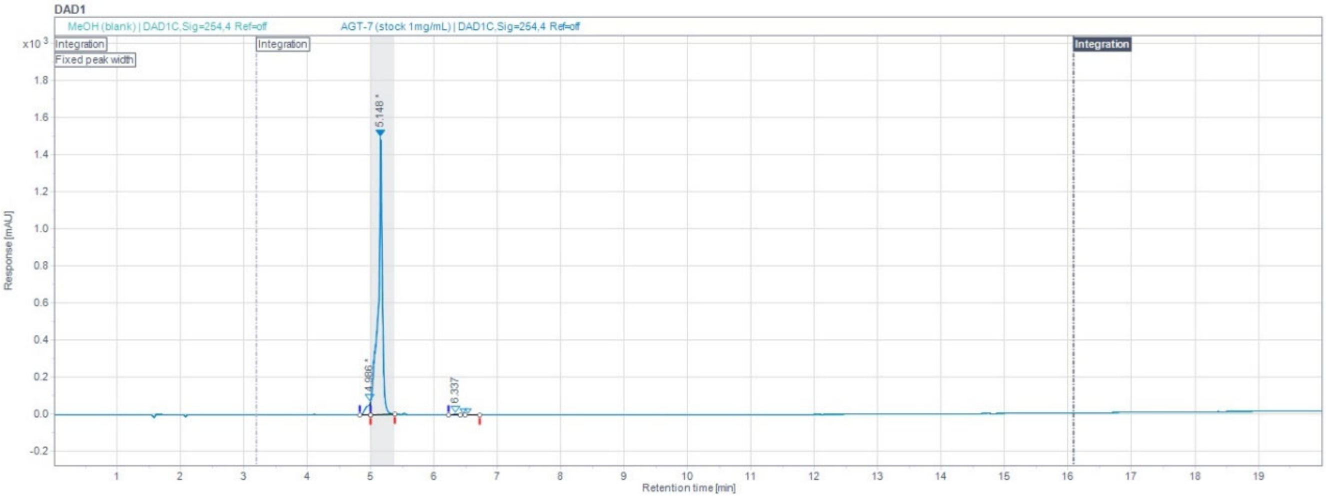

Figure S1: Illustration of HPLC chromatogram conducted for the clarification of the purity of the compound. The desired peak appears at 5,15 min and signifies purity of 95.3%.

Table S1: Peak analysis of the analytical HPLC that indicates the retention times and the purity of the compound.

Peak	RT (min)	Area (mAU·s)	Area%	Height (mAU)	Height%	Start time (min)	End time (min)
1	4,986	288,754	4,202	64,604	4,13	4,817	4,987
2	5,148	6546,913	95,275	1491,659	95,32	4,987	5,371
3	6,337	24,036	0,35	5,622	0,36	6,228	6,404
4	6,451	5,052	0,074	1,471	0,09	6,404	6,482
5	6,525	6,82	0,099	1,524	0,1	6,482	6,719

NMR Characterization of tert-butyl (4-(bis(pyridin-2-ylmethyl)amino)butyl)carbamate

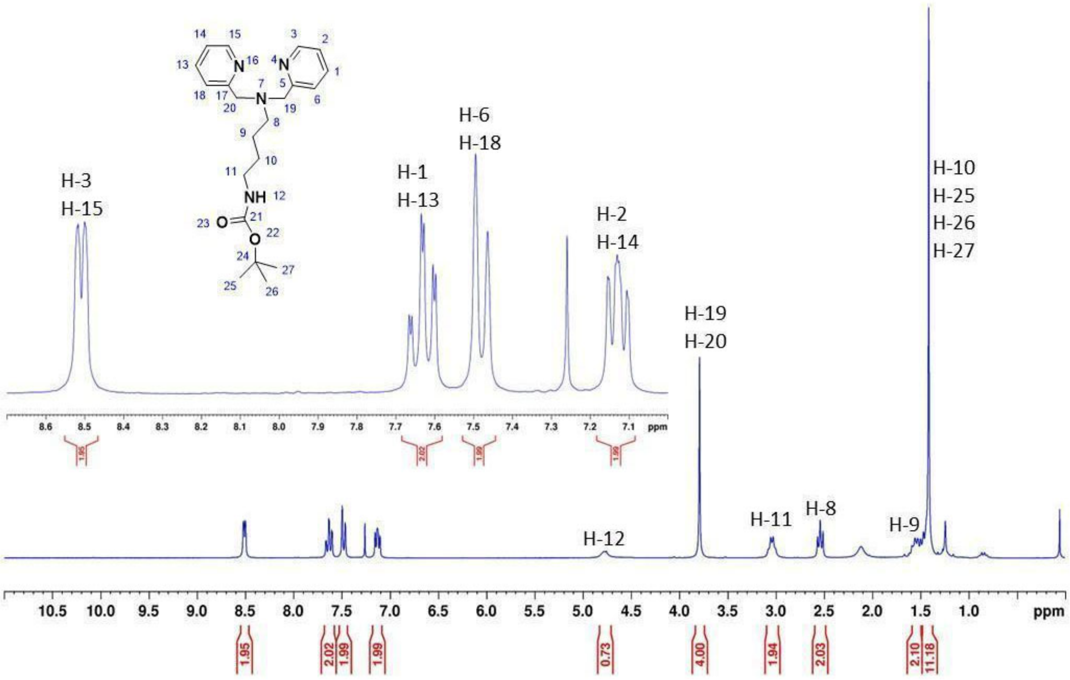

Figure S2: ¹H-NMR spectra of the tert-butyl (4-(bis(pyridin-2-ylmethyl)amino)butyl)carbamate.

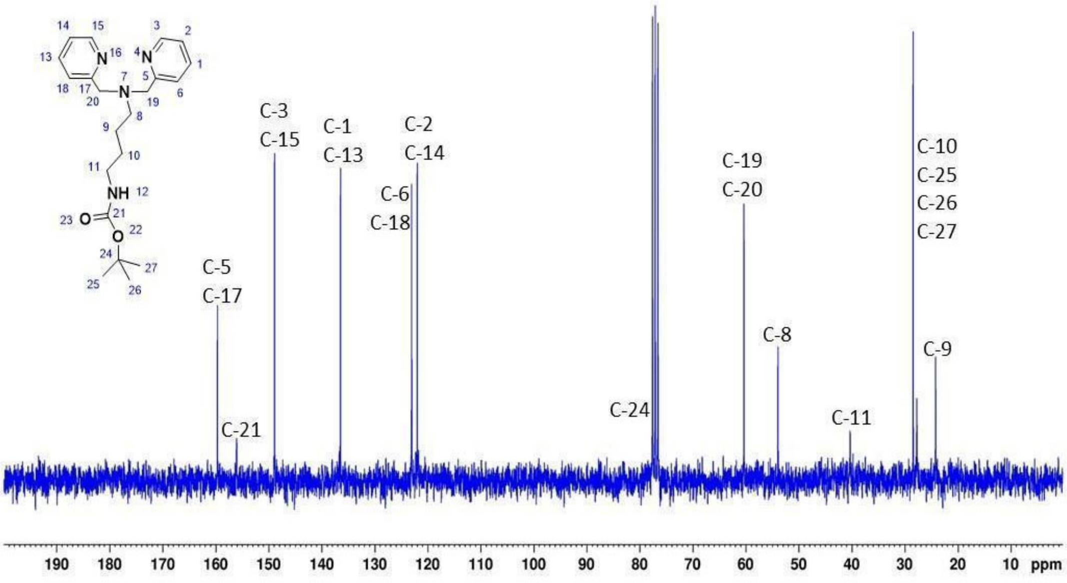

Figure S3: ¹³C-NMR spectra of the tert-butyl (4-(bis(pyridin-2-ylmethyl)amino)butyl)carbamate.

NMR Characterization of N1,N1-bis(pyridin-2-ylmethyl)butane-1,4-diamine

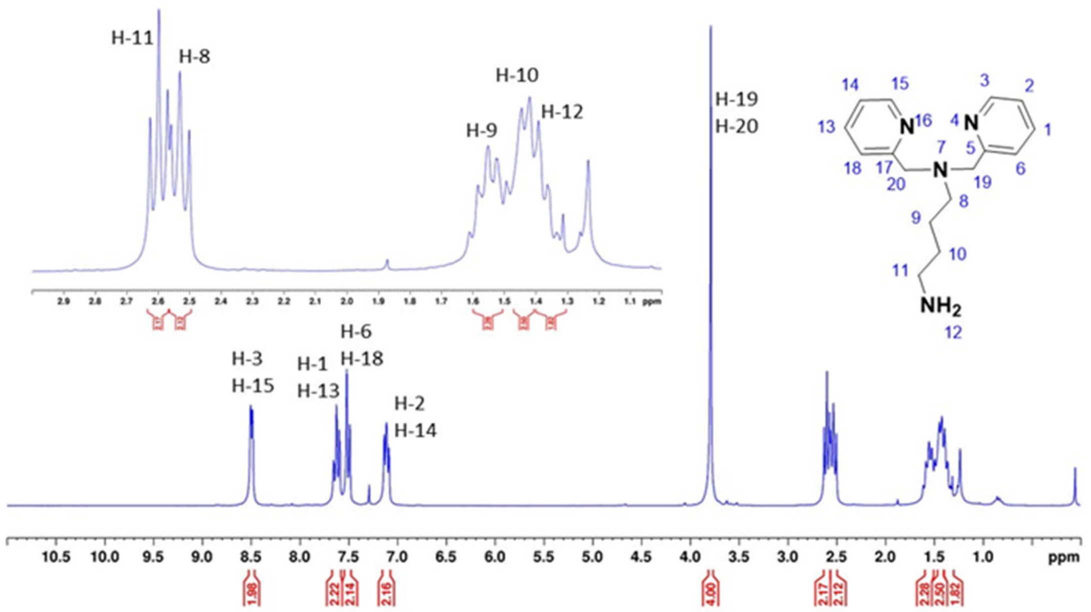

Figure S4: ^{1}H-NMR spectra of the N1,N1-bis(pyridin-2-ylmethyl)butane-1,4-diamine.

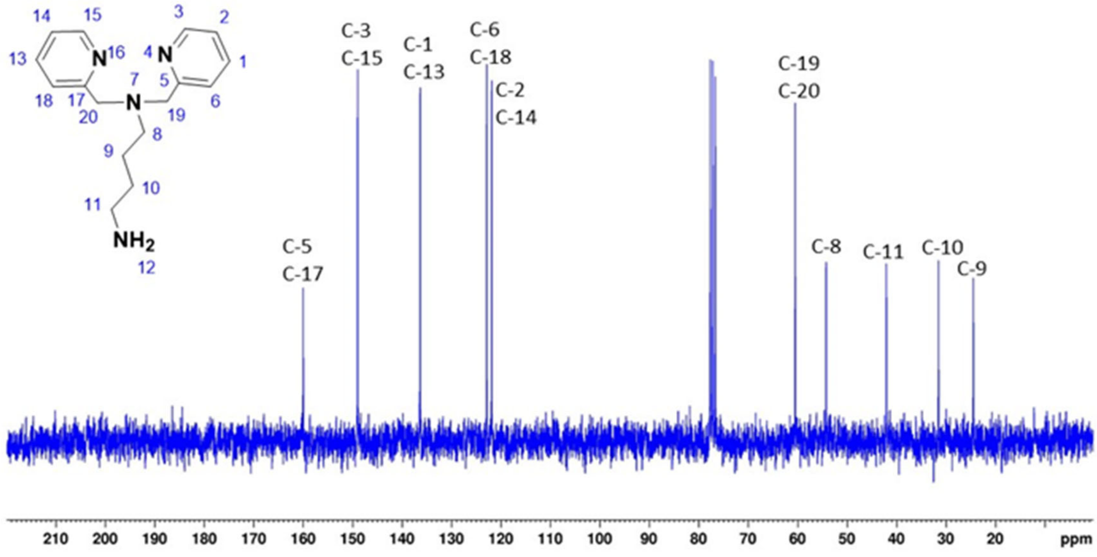

Figure S5: ^{13}C-NMR spectra of the N1,N1-bis(pyridin-2-ylmethyl)butane-1,4-diamine.

Purification by preparative HPLC of compound AGT-7

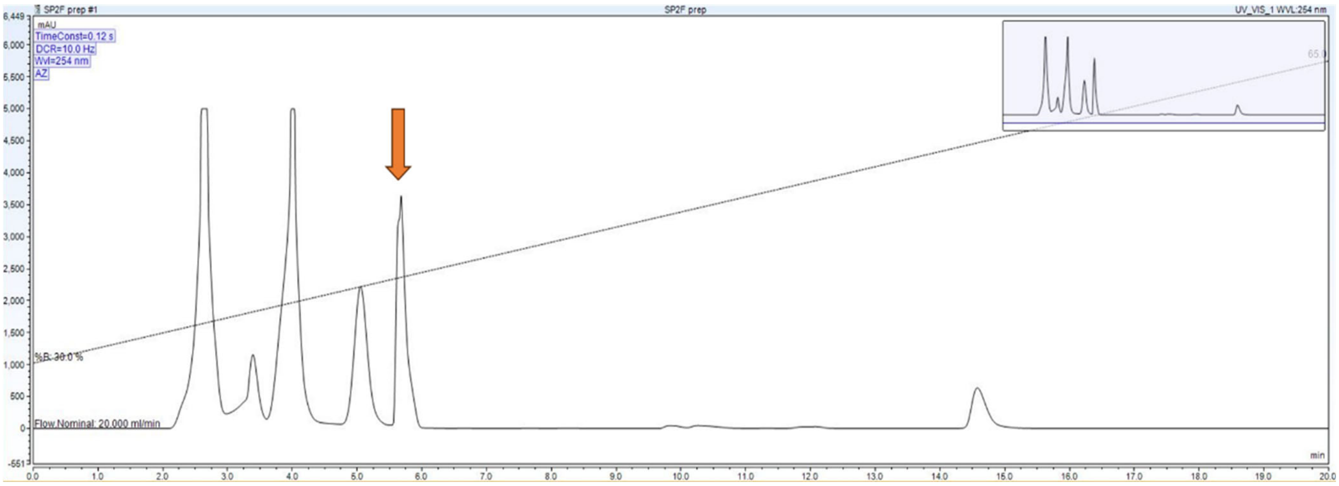

Figure S6: Illustration of an HPLC chromatogram conducted for the purification of the compound. The desired peak, indicated by the arrow, appears at 40% acetonitrile.

NMR and MS-Based Characterization of AGT-7

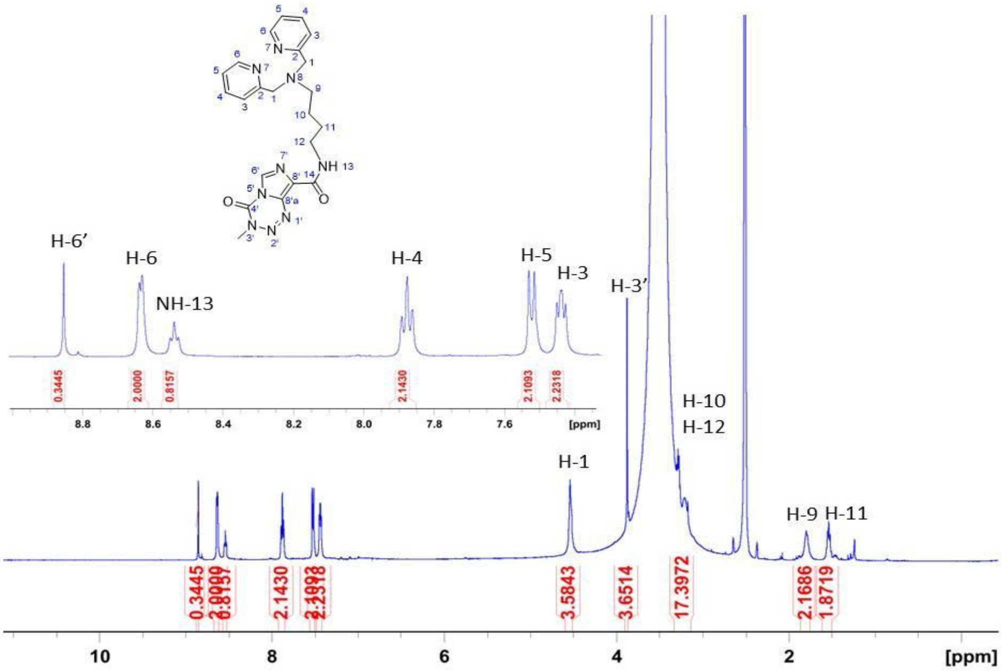

Figure S7: ^{1}H-NMR spectra of **AGT-7**

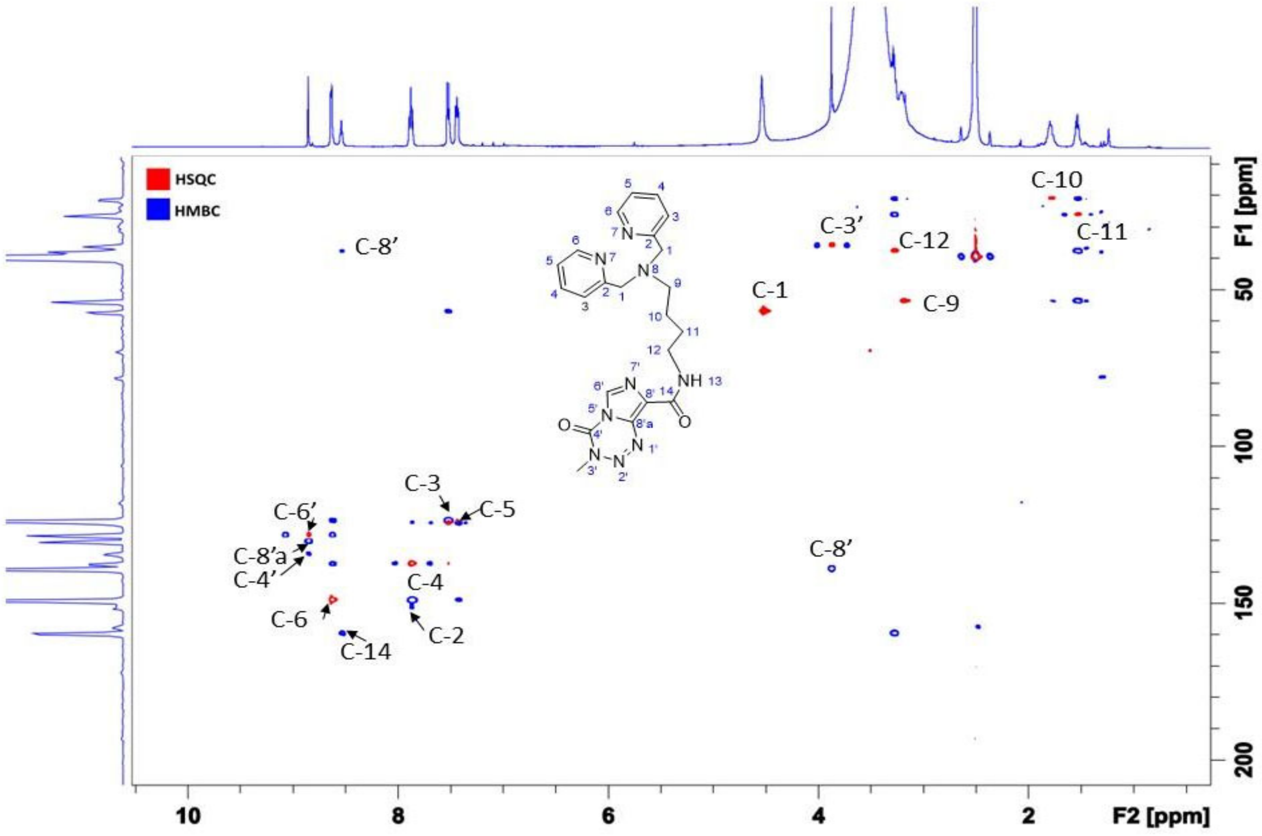

Figure S8: HSQC (red) and HMBC (blue) overlay spectra of the compound **AGT-7**.

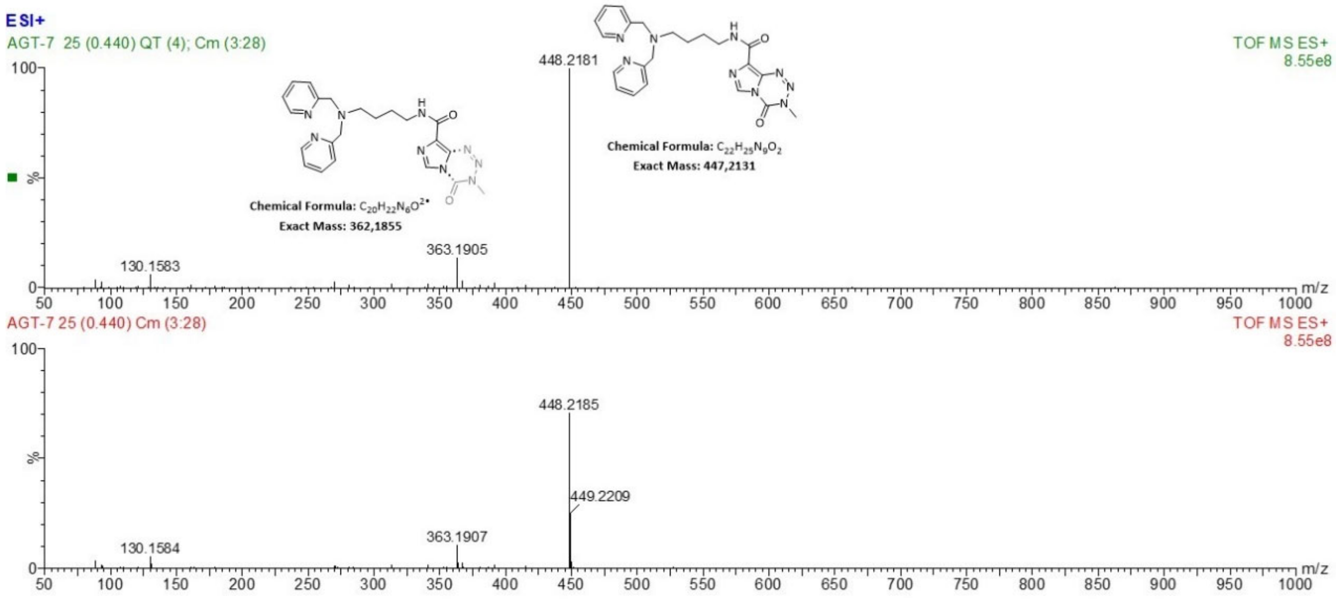

Figure S9: MS-spectra of **AGT-7** (Chemical Formula: $C_{22}H_{25}N_9O_2$) with the calculated m/z [M+H] 447,2131. The spectra also displayed the identified fragments in positive ionization mode. The image below describes the spectrum that was recorded, and the image above describes the spectrum after the TOF Transform process where it both de-isotopes masses and realigns to a single charge state mass axis.

References

1. Kimmel, C.B.; Patterson, J.; Kimmel, R.O. The Development and Behavioral Characteristics of the Startle Response in the Zebra Fish. Dev. Psychobiol. **1974**, 7, 47–60.
2. OECD, 2010. Short Guidance on the Threshold Approach for Acute Fish Toxicity Testing. Series on Testing and Assessment No. 126. OECD, Paris, France. Available at: <www.oecd.org>.
